# Investigating the introduction of porcine epidemic diarrhea virus into an Ohio swine operation

**DOI:** 10.1186/s12917-015-0348-2

**Published:** 2015-02-15

**Authors:** Andrew S Bowman, Roger A Krogwold, Todd Price, Matt Davis, Steven J Moeller

**Affiliations:** The Ohio State University College of Veterinary Medicine, 1920 Coffey Road, Columbus, OH 43210 USA; USDA, APHIS, Veterinary Services, Pickerington, OH 43147 USA; North Central Veterinary Service, Sycamore, OH 44882 USA; Hord Livestock Company, Bucyrus, OH 44820 USA; The Ohio State University College of Agriculture, Columbus, OH 43210 USA

**Keywords:** Feed, PEDV, Swine

## Abstract

**Background:**

Porcine Epidemic Diarrhea virus (PEDV) is a highly transmissible coronavirus that causes a severe enteric disease that is particularly deadly for neonatal piglets. Since its introduction to the United States in 2013, PEDV has spread quickly across the country and has caused significant financial losses to pork producers. With no fully licensed vaccines currently available in the United States, prevention and control of PEDV disease is heavily reliant on biosecurity measures. Despite proven, effective biosecurity practices, multiple sites and production stages, within and across designated production flows in an Ohio swine operation broke with confirmed PEDV in January 2014, leading the producer and attending veterinarian to investigate the route of introduction.

**Case presentation:**

On January 12, 2014, several sows within a production flow were noted with signs of enteric illness. Within a few days, illness had spread to most of the sows in the facility and was confirmed by RT-PCR to be PEDV. Within a short time period, confirmed disease was present on multiple sites within and across breeding and post weaning production flows of the operation and mortality approached 100% in neonatal piglets. After an epidemiologic investigation, an outsourced, pelleted piglet diet was identified for assessment, and a bioassay, where naïve piglets were fed the suspected feed pellets, was initiated to test the pellets for infectious PEDV.

**Conclusions:**

The epidemiological investigation provided strong evidence for contaminated feed as the source of the outbreak. In addition, feed pellets collected from unopened bags at the affected sites tested positive for PEDV using RT-PCR. However, the bioassay study was not able to show infectivity when feeding the suspected feed pellets to a small number of naïve piglets. The results highlight the critical need for surveillance of feed and feed components to further define transmission avenues in an effort to limit the spread of PEDV throughout the U.S. swine industry.

## Background

Porcine epidemic diarrhea virus (PEDV) is a coronavirus of the genus *Alphacoronavirus.* Disease from PEDV is characterized by vomiting, anorexia, and watery diarrhea in swine. The virus is particularly deadly for neonatal pigs for which malabsorption and dehydration [1-3] can result in mortality rates approaching 80%-100% [2,4]. Disease caused by PEDV is clinically indistinguishable from transmissible gastroenteritis virus and cannot be diagnosed on presentation alone [4]. Because attempts at virus isolation have only resulted in limited or temporary success, with virus isolation rates as low as 4% [5], diagnosticians heavily rely upon RT-PCR tests to directly detect viral nucleic acid and diagnose PEDV.

PEDV was first identified in Belgium in 1978 and in the 1980s and 1990s, PEDV was found throughout Belgium, England, Germany, France, the Netherlands, and Switzerland [6]. Since the European emergence, PEDV has affected the pork industries in Philippines, South Korea, and China [7]. In May 2013, the United States confirmed the first cases of PEDV on farms in Iowa and Indiana [2], after which the virus spread quickly throughout the country. While the mode of PEDV introduction to the U.S. remains unknown, comparison of available sequence data indicates the PEDV strains detected in the Unites States have an ancestry linked to PEDV strains detected in China. At the end of 2013, sequenced U.S. strains had greater than 99.0% sequence identity and several strains shared unique nucleotides with a Chinese PEDV strain isolated in the Anhui Province (AH2012) [2,3]. Unexpected genetic similarity of U.S. PEDV strains to a bat coronavirus isolated in southeastern China may provide evidence for the role of cross-species transmission in the development of emergent strains that spread to the United States [3].

Transmission of PEDV occurs via the fecal-oral route [7] and fecal contamination of fomites may play a role in the introduction of the virus to swine. An investigation of 575 livestock trailers at 6 harvest facilities in the United States showed that all truck drivers stepped into the harvest facility at least once, and the proportion of PEDV contaminated trailers increased from 6.6% before unloading to 9.2% after unloading [8]. These data indicate that contaminated transport vehicles and personnel could be associated with the rapid spread of the virus throughout the US. At present, PEDV prevention and control in the U.S. are heavily dependent on biosecurity procedures.

While transportation equipment might play a role in the spread of PEDV, on-farm investigations into several PEDV outbreaks in the United States have indicated that contaminated feed could be a pathway of viral introduction; however, scientific support of this route is regularly debated. Dee *et al*. showed that material collected from the inside of feed bins during a PEDV outbreak was infectious when concentrated and inoculated into pigs [9]. One Canadian report showed spray-dried porcine plasma, a component used in some swine feed, was infectious to pigs, but the complete feed containing spray-dried porcine plasma was not infectious in an experimental setting [10]. On the other hand, a study team has provided contrary evidence with an unsuccessful attempt to infect pigs with spray-dried porcine plasma and data that indicates PEDV is inactivated during spray-dried porcine plasma production process [11,12].

In January 2014, an outbreak of PEDV was confirmed in a multi-site, multiple flow swine operation in Ohio. After a thorough epidemiologic investigation, contaminated feed was identified as the likely source of pathogen introduction, a finding supported by a positive RT-PCR result from testing the feed source. Of note, RT-PCR detects viral RNA, and thus can only confirm the presence of viral nucleic acid in a sample, not necessarily presence of viable and infectious virus. Since PEDV isolation is very difficult in numerous testing and research laboratories, virus isolation attempts from feed pellets could not be relied on to detect viable, infectious virus. Consequently, a bioassay was initiated where samples of feed cryopreserved by the attending veterinarian during the outbreak were later fed to naïve piglets in an attempt to demonstrate feed infectivity. This report will discuss the aforementioned epidemiologic investigation and subsequent bioassay findings.

## Case presentation

**Figure 1:**
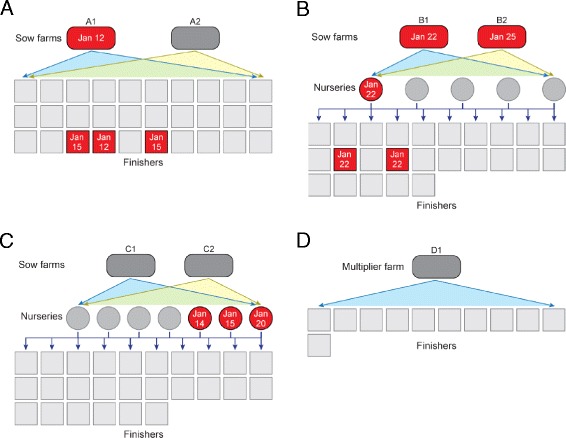
**A schematic representation of the pork production system with each of the four production flows represented in separate panels.** Panels **A**, **B**, and **C** illustrate the three separate multi-site, farrow-to-finish production flows within the pork production system which are referred to as flows **A, B**, and **C** respectively. Weaned pigs from flow **A** are placed into wean-to-finish barns, whereas pigs from flows **B** and **C** are weaned into nursery facilities and later moved to finishing barns. Production flow **D**, as represented in Panel **D**, is a multiplier herd with a single breed-wean site. Weaned pigs from flow **D** are raised in gilt developer units or wean-to-finish barns. Production sites where porcine epidemic diarrhea virus (PEDV) was detected during the outbreak are shaded red and the date of PEDV detection is listed.

The Ohio swine operation (Figure 1), consisting of 3 multi-site, farrow-to-finish production flows (referred to as flows A-C, each having two breed-wean sites) and a multiplier herd (referred to as D, with a single breed-wean site) had no prior cases of PEDV and was determined to have effective biosecurity measures in place evidenced by the absence of Porcine Reproductive and Respiratory Syndrome Virus (PRRSV) during more than the prior seven years. Routine oral fluid testing of pigs in flow B on January 8, 2014 and surveillance testing in flow C in November and December 2013 were all negative for PEDV. At time of weaning, pigs move from breed-wean premises to wean-to-finish barns for flow A and to nursery facilities and then finisher sites for flows B and C. Weaned pigs from flow D, the multiplier herd, are raised in gilt developer units or wean-to-finish barns.

### Disease outbreak

On the morning of January 12, 2014, four lactating sows from the one of the breed-wean units in flow A (unit A1) were noted with vomiting and diarrhea. The illness spread rapidly and by 4:00 pm, 80 litters showed signs of diarrhea, vomiting, and dehydration. Within a few days, 80% of sows on the site showed similar clinical signs. Fecal samples taken January 15, 2014 were positive for PEDV using RT-PCR. Forty-two percent mortality was observed in piglets in the A1 farrowing unit. Beyond the sow unit, a wean-to-finish barn in flow A that received pigs on January 10^th^ from both flow A breed-wean units (A1 and A2) reported loose stools on January 12^th^ and had confirmation of PEDV with RT-PCR positive fecal samples collected the same day. Also within flow A, one wean-to-finish barn that was filled with piglets from both flow A breed-wean units (A1 and A2) on January 10^th^ and 12^th^ had fecal samples test PEDV RT-PCR positive on January 15, 2014. In addition, on January 15^th^, a third wean to finish barn that received pigs from both flow A sow farms on January 9^th^ had fecal samples test PEDV positive. A schematic of flow A is shown in Figure 1A.

While no PEDV-like disease was observed in either breed-wean units in flow C, pigs in 3 nurseries within flow C did test PEDV RT-PCR positive between January 14^th^ and January 20^th^, 2014 (Figure 1C). On January 22, 2014 one breed-wean unit within flow B (B1) began experiencing PEDV-like disease. On that same day, an oral fluid sample from one of the nurseries in flow B that received pigs from both flow B breed-wean units (B1 and B2) on January 15^th^, 17^th^, and 20^th^, 2014 tested PEDV PCR positive (Figure 1B). Also on January 22^nd^, two finishing barns in flow B had a PEDV PCR positive oral fluid test. By January 25^th^, the second breed-wean unit in flow B (B2) was also experiencing the disease. Overall, mortality among neonatal piglets was close to 100% in flow B.

### Epidemiological investigation

Five American Association of Swine Veterinarians (AASV) PEDV questionnaires were completed by a USDA epidemiologist and an Ohio Department of Agriculture Veterinary Medical Officer in conjunction with swine operation representatives and the operation’s local veterinarian. Several potential pathways of pathogen introduction to the swine operation, including human introduction, delivery of contaminated supplies, aerosol spread, contaminated pig transport vehicles, and contaminated feed or feed ingredients were considered and evaluated.

It is unlikely PEDV was introduced to the operation by visitors or workers. There were no foreign visitors, and no employees had visited foreign countries within 10 days of the outbreak. Nor did any employee have swine at their place of residence or associated farm enterprises. All swine operation employees and non-employee contractors follow meticulous biosecurity procedures to enter a facility, and movement of people from one facility to another within the same day is limited to production managers only, which typically occur only within the same flow. Effectiveness of the biosecurity measures in place was evidenced by the absence of PRRS cases for over seven years.

Veterinary, vaccine, and semen supplies delivered by supply vendors were also considered as a potential source of PEDV introduction to the swine operation, but were subsequently ruled out as likely sources for several reasons. First, supplies are delivered to buildings separate from the swine housing areas and they are not shared among different flows. Disease, however, broke out separately in geographically and personnel isolated units from 3 different flows. In addition, supplies were disinfected in a fume chamber within the enclosed room whereby the incoming materials were placed on an elevated metal grate and a mister system applied a quaternary ammonium/glutaraldehyde combination disinfectant (Synergize, Preserve International, Reno, NV) and allowed to stand for 15 minutes before entry into site. This practice was considered to greatly reduce the likelihood of contaminated supplies as the potential route of PEDV introduction.

Airborne spread could be considered with PEDV [13], as coronaviruses classified within the same group as PEDV (group 1 coronaviruses) include those that cause enteric or respiratory infection. Porcine respiratory coronavirus is a mutant of transmissible gastroenteritis virus and is an example of a group 1 coronavirus that is spread through droplets and aerosols [14]. Aerosols or droplets are unlikely to be responsible for the spread of disease on this swine operation because most units are not located geographically close to each other, and disease broke on multiple separate units from 3 separate flows across a period of 13 days. Additional factors that could be involved in virus transmission such as water supply and shavings used during transport of young pigs are not probable because pigs on all sites have equal exposure to these factors but not all sites were involved in the outbreak.

Lowe *et al.* have shown that contaminated transport vehicles are likely to be involved in rapid spread of PEDV because it is common to transport pigs to harvest facilities on vehicles that have not been disinfected between loads [8]. In relation to the swine operation involved in this outbreak, it is improbable that contaminated vehicles were involved. First, the operation is closed, meaning no swine are brought on site unless they are owned by the entity and managed under the stringent biosecurity procedures displayed by the operation. Second, the operation maintains 3 truck wash facilities where written protocols are followed to thoroughly clean, wash and disinfect all company trucks. The production company regularly audits the truck wash facilities and was actively testing trucks, trailers, drying equipment, and wash bays for PEDV; all samples taken prior to the outbreak and during the first week of the outbreak were negative for PEDV. Cull animals are hauled on cull-only trailers controlled by the production system and are washed, disinfected, dried and inspected prior to use. Cull animals are transferred to a neutral location where the animals are transferred onto a third party hauler’s washed and disinfected trailer for market delivery. Finisher trucks are not disinfected at company truck washes but rather at truck washes external to the production system. Given lack of production system control over these external truck washes, finishing trucks are perceived as a higher biosecurity risk to the operation; however, trucks transporting finisher swine go only to harvest facilities and do not come in contact with pigs or sows from breed-wean units within the operation.

This operation primarily uses feed produced by the operation’s on-site feed mill, with exception of an outsourced starter pellet fed to piglets at the time of weaning and a commercial meal mix used to start nursery pigs on pellets. It was determined that neither feed supplied by the operation’s own mill, nor the commercial meal mix were likely to be involved in the transmission of PEDV to pigs on the operation. Prior to the outbreak, the same internal feed ingredients and commercial meal mix had been used with no ill effects. Also, internal feed ingredients were used across all swine units within the operation, but not all swine units were involved in the outbreak.

Because the timing of the outbreak seemed to coincide with the switch to a new source of starter pellet feed, the attending veterinarian and farm officials suspected the new supplier’s starter pelleted diet was the source of pathogen introduction. Results of the epidemiologic investigation indicated PEDV genetic material presence in the starter feed, validating this suspicion.

### Starter feed pellets as a source of pathogen introduction

Starter feed pellets from the new supplier were offered to piglets in the A1 farrowing facility during the week of January 6, 2014. By January 12, 2014, clinical signs of PEDV were present among sows and piglets in the A1 facility. Starter feed pellets were subjected to standard biosecurity procedures to enter into the facility. In short, feed bags are placed into clean bins from the facility, and bins loaded with feed are disinfected in a fume chamber as they are transferred into the facility. Following this protocol eliminates contamination from the outer surface of the bag as the source and indicates feed ingredients are likely the source of contamination. Supporting the introduced pellets as a likely source, the A2 breed-wean site within the same flow, which never received the new supply of feed pellets, remained PEDV negative. Pigs in one nursery in flow B (BN1), 3 nurseries in flow C (CN5, CN6 and CN7 west barn), and one wean-to-finish unit in the multiplier herd (D) were also started on the new supplier’s feed pellets. All of these sites were subsequently found to be PCR positive for PEDV except the flow D wean-to-finish unit. It is thought that differences in pellet storage conditions may account for this inconsistency from the flow D unit. Flows B and C store their feed pellets in a room separate from the barn. During winter, these rooms are estimated to be at 40°F (approximately 4°C). The units in flow D store their pellets within the barn where temperatures are around 80°F (approximately 27°C). Storage in higher temperatures may have inactivated the virus. This hypothesis is supported in a study by Jung and Chae where storage of fecal samples at temperatures 21°C and greater resulted in a decline in PEDV nucleic acid detection by RT-PCR when compared to those stored at 4°C [15].

Another inconsistency was that two contract finisher facilities in flow B and both B flow breed-wean units also broke with disease or tested positive for PEDV, even though these facilities did not receive the new supplier’s pellets. Because PEDV is highly transmissible, spread of disease from the units where it broke to the breed-wean units by human error cannot be ruled out. Of note, the same person does chores at the B1 farrowing unit and the BN1 nursery where pigs were fed the implicated pellets. Also, the two flow B finisher facilities had just received pigs and do share a person who does chores between them, but that person did not have direct contact with any of the sow units.

Along with the timing of the outbreak that coincided with the switch to new supplier’s feed pellets, strong evidence for these feed pellets as the source of the outbreak comes from PCR testing of the new supplier’s pellets. Pellets from the BN1 nursery tested PEDV positive by RT-PCR on January 17, 2014 (C_t_ value = 32.95). Additionally, three lots of feed pellets from BN2 nursery, which had been cleaned and disinfected and was empty of pigs at the time, tested positive for PEDV by RT-PCR (mean C_t_ value = 32.58). Pigs that had left this nursery and were now in a finisher facility tested negative for PEDV, showing this nursery had been negative for PEDV while it was housing pigs. Similar findings result from testing of units within flow C. Feeder pigs that left the facility at the end of December and beginning of January were tested and found to be PEDV negative, confirming that no disease was present prior to January 12. One of the afflicted flow C nursery sites (CN7) consists of 2 barns labeled east and west. CN7 west barn received new pigs, pellets from the new supplier, and subsequently broke with PEDV. At the same time, the CN7 east barn housed PEDV negative pigs weighing approximately 50 lbs. from the previous placement. These pigs stayed PEDV negative after moving offsite all the way through marketing. Interestingly, CN7 west barn had pellets from both the old and new suppliers in the barn at the time of the outbreak. The test results showed swabs taken on the outside of both old and new suppliers’ pellet feedbags were PEDV RT-PCR positive (mean C_t_ value = 31.93). Therefore, pellet samples were collected with care to avoid contamination from the exterior of the bags. Briefly, the top 25 cm of the feedbags were wiped with a 0.52% solution of sodium hypochlorite. Bags were then opened by cutting the top of the bag off with a scalpel to ensure a minimum risk for potential dust contamination. Feed samples were retrieved from the center of each bag by the attending veterinarian who was wearing a sterile obstetrical sleeve. The samples were placed into a sterile plastic bag, sealed, and submitted for testing. PEDV RT-PCR was positive (mean C_t_ value = 33.34) for the new supplier’s pellets and PEDV RT-PCR negative for the old supplier’s pellets. These results were interpreted to mean that the exterior of the feedbags had become contaminated with PEDV in the barn during the outbreak; however, since PEDV was detected in the interior of the unopened bags of the new supplier’s pellets, PEDV contamination of this feed had to occur prior to delivery at the barn.

Building on the RT-PCR results from new supplier’s feed pellets on the swine operation, back-up pellets of the same lots at the new supplier’s manufacturing facility also tested RT-PCR positive (mean C_t_ value = 32.97). Testing of individual ingredients at the new supplier’s facility yielded several positive results. Strong evidence implicating pellets from the new supplier as the contamination source based on the PEDV RT-PCR positive results is firmly supported by findings of the epidemiologic investigation. The epidemiologic investigation also concluded that virus isolation from pellets would be critical evidence that the pellets caused the outbreak.

### Bioassay design

Since PEDV is very difficult to isolate, a bioassay was initiated to determine if the pellets in question could infect naïve piglets. During the outbreak at the swine operation, the attending herd veterinarian aseptically collected aliquots (as described above) of the RT-PCR positive pelleted feed from the farm and mixed them with sterile phosphate buffered saline to make a mash. These moistened, mash aliquots were stored at −20°C until the bioassay could be performed.

Ten, 10-day-old pigs, were obtained from a commercial sow herd. Sows from the source herd, the facility where the bioassay was performed, and the piglets were all confirmed to be negative for PEDV by RT-PCR at the start of the bioassay. Serum, collected from the pigs prior to leaving the source farm, tested negative for PEDV antibodies using an indirect immunofluorescence assay. During a 108 hour acclimation period, pigs were fed a commercial swine starter feed and rectal swabs from the pigs, feed samples, and environmental swabs were all collected on a daily basis. Following the acclimation period, the pigs were provided ad libitum access to the RT-PCR positive mash along with dry pellets from the same lot for 7 days, and observed for clinical signs of PEDV. Feed samples, environmental swabs, and rectal swabs were collected each day of the study. After 7 days, the pigs were euthanized and intestinal tissues were submitted for diagnostic testing.

### Bioassay results

The environment, starter feed, and pigs were PEDV negative using RT-PCR prior to the study and during the 108 hour acclimation period. Mash aliquots and pelleted feed obtained from the swine operation site tested weakly PEDV positive with RT-PCR during the 7 day study (mean Ct = 36.5). Pigs were observed to be very healthy during the bioassay and no clinical signs of disease were observed in the pigs during the bioassay. Environmental and rectal swabs collected daily during the study were negative for PEDV using RT-PCR. Microscopic examination of intestinal tissues collected from the piglets at the end of the study revealed no significant morphologic lesions.

Although the bioassay results did not confirm the feed pellets in question were infectious, feed cannot be ruled out as the cause of this outbreak. In the present study, the sensitivity of the bioassay was limited by the amount of feed the individual pigs and the small number of pigs collectively could consume during the trial period. Even if infectious virus was present in the feed used for the bioassay, the mean C_t_ value of 36.5 indicates it would be present at very low concentration. In addition, the pigs evaluated appeared healthy, with what was likely limited disease challenge resulting in little immune or digestive system compromise. In a field setting where there are thousands of pigs consuming tons of feed, and known, observable presence of unthrifty pigs with potentially compromised digestive or immune systems, it is conceivable that a very small amount of infectious PEDV in a food source would be capable of initiating an outbreak that would rapidly spread through the population of susceptible animals. In addition, the present bioassay portion of the study may have been hindered by the 28 day lag from the time the feed was manufactured and the initiation of the bioassay. The time lag likely decreased the viability of any infectious PEDV that was present in the feed at the time of delivery to the farm.

## Conclusions

Because the timing of this outbreak coincided with a switch to new out-sourced feed pellets and due to the strong evidence provided by PEDV positive RT-PCR results of these feed pellets at both the swine operation and the supplier, it is believed that contaminated feed pellets were the source of this outbreak. A study reported subsequent to completion of the present study proved that contaminated feed can serve as a vehicle to transmit PEDV to naïve pigs [9]. The results of the epidemiologic investigation, proof of concept by other investigators and the presence of PEDV RNA from unopened bags of feed all support feed as the source of the outbreak. The inability of a bioassay to prove the feed pellets were infectious after the outbreak occurred must be considered, but the low sensitivity of this assay does not rule out feed as possible source. The results of the present and other studies demonstrate the need for strict biosecurity practices and thorough testing for feed and feed ingredients used in the pork industry for which, PEDV outbreaks can cause devastating financial losses and PEDV surveillance and prevention efforts are of the utmost importance.
